# An absorptive capacity-based systems view of Covid-19 in the small business economy

**DOI:** 10.1007/s11365-021-00753-7

**Published:** 2021-06-05

**Authors:** Rosa Caiazza, Phillip Phan, Erik Lehmann, Henry Etzkowitz

**Affiliations:** 1 Parthenope University, Naples, Italy; 2 Johns Hopkins Carey Business School, Baltimore, MD USA; 3 Augsburg University, Minneapolis, MN USA; 4 Stanford University, Stanford, CA USA

**Keywords:** Covid-19, Entrepreneurship, SME, M13, I10, I18, L26

## Abstract

According to Johns Hopkins University, by December 2020, more than 78 million SARS-COV-2 (Covid-19) cases have been reported with more than 1.7 million deaths, out of which more than 300 thousand were in the U.S. alone. No country on earth has been untouched by the preemptive creation of a global recession to combat a global disease. Covid-19 has disrupted supply chains, consumption patterns, and business models in a multitude of industries which include a large share of small and medium enterprises (SMEs). SMEs account for the largest share of employment in market-based economies so any discussion of the economic impact of Covid-19 is incomplete without the SME sector. The purpose of this paper is to explore a systems perspective of the Covid-19 pandemic using the absorptive capacity construct.

## Introduction

In December 2019 officials in Wuhan, China reported the first case of the novel coronavirus or SARS-COV-2 (Murrey, 2020; Kuckertz et al., 2020). By December 2020, that number grew to more than 78 million Covid-19, the disease caused by SARS-COV-2, cases and 1.7 million deaths, of which more than 300 thousand were in the US.^1^ No country has been untouched, accompanied by the unprecedented preemptive creation of a global recession to combat a global disease (Murrey, 2020).

The emerging literature on the impact of public infection control measures has discussed such effects as social disorder, heightened health disparities in minority communities, increased mortality among the aged, and disproportionate hardship in rural labor markets (McKibbin & Fernando, 2020). These effects have disrupted supply chains, consumption patterns, and business models in such industries as transportation, real estate, manufacturing, tourism, and consumer retail. These industries include a large share of small and medium enterprises (SMEs), which also account for the largest share of employment in most economies. Moreover, the specific combination of competitive, environmental, technological, and geographical factors implies that the relationship between Covid-19 and SMEs is a multi-level systemic phenomenon (Caloghirou et al., 2020; Sveiby, 2012).

In this paper, we discuss a systems view of the impact of Covid-19 on SMEs. To address the limitation of systems models, which are atheoretical descriptions of dynamic phenomena, we ground our discussion on the construct of absorptive capacity. Cohen & Levinthal (1990) define absorptive capacity as an organization’s capacity to recognize the value of new external knowledge, assimilate and to create economic value from it. The construct has been applied in psychology (Edmondson, 2001), sociology (Levitt & March, 1988), economics (Cockburn & Henderson, 1998), and political science (Wegloop, 1995) to explain adaptation and innovation by individuals, organizations, and institutions (Omorede, 2020; Llopis et al., 2015).

The literature on crisis management in organizational research is summarized in a seminal paper by Pearson & Clair (1998), in which they point out that the research in crisis management is typified in the following way. First research on crisis management is multidisciplinary, relying on work in psychology, sociology, technology, and economics to describe and explain the impact of a crisis event on organizations. Second, the most informative research relies on systems models although there continues to be work from a single disciplinary perspective, e.g., social psychology, that draw from other disciplines to fill out conceptual gaps (Vegetti & Adăscăliţei, 2017). Third, and more importantly, they summarize the research in crisis management from an organization level unit of analysis. Accordingly, crises are salient to an organization if they materially affect strategy and structure. Hence, the authors define crisis management strategy as one that steers an organization away from the crisis or mitigates the worse effects of the crisis on the organization’s functioning. In their view, and that of other researchers, crisis management comprises the decisions and processes that returns an organization to its pre-crisis equilibrium.

More recently, especially in light of the Covid-19 pandemic, scholars have explored the relationship between crises and individual decision-making behaviors (Shoss et al., 2021). Using event systems theory, they show that perceptions of the nature and severity of the Covid-10 crisis are negatively related to helping behaviors, i.e., altruist actions targeted toward another, at the individual level of analysis. To the extent that helping behaviors are necessary for business and societal recovery after economic crises and natural disasters, such findings could be concerning although the evidence does not rise up to the level of a general conclusion.

Our paper extends the systems approach of Pearson & Clair (1998) by discussing the multi-*level* effects of a crisis on a class of organizations (SMEs) and the latter’s responses. The question of entrepreneurial responses to economic crises and natural disasters has been an active area of research (c.f., Santos et al., 2021). We build on this line of inquiry by starting from the assumption that recovery from the crisis is not necessarily a return to a pre-crisis equilibrium. Instead, give the scale and severity of the pandemic, we posit on the long lasting institutional and structure (consumption patterns) changes that point to a reordering (in the Schumpeterian sense) of the opportunities confronting SMEs.

We argue that while crises can negatively affect entrepreneurial intentions, as previous studies have suggested, they create resource voids that represent opportunities for starting new businesses or promoting business expansion (Bullough et al., 2014; Doern et al., 2016). Social ‘bads’ represent unmet needs that, in the hands of entrepreneurs, translate into opportunities for value creation. In a sense, new venture creation in the shadow of the pandemic illustrates entrepreneurship in its purest form, in which the creation of new ventures arises from the needs of society, dissolving the customary divide between social and commercial interests (Williams & Sheperd, 2016; Grube & Storr, 2018).

Our paper proceeds as follows. We first provide background on the disease etiology and the ensuing public health mitigation strategies. This is because the primary impact of the pandemic is on human health, which is the predicate for stable labor and consumption markets, and the way we will understand the SME economy. Next, we articulate an absorptive capacity systems theory of the crisis by reviewing the concept of absorptive capacity and positioning it within the systems perspective. We then apply the theory to the macro, micro and individual levels of analyses. In light of our discussion, we reflect on the policy interventions so far and SME recovery to come. We conclude by discussing the limitations of our paper and future directions for research.

## Background

By most accounts, the first recorded case of SARS-COV2 human infection, the virus that causes Covid-19, was in the city of Wuhan, China in December 2019 (Hua & Shaw, 2020). By January 2020, Wuhan city was locked down, a public health intervention that became the standard model for infection control. The lock down orders post-dated the Chinese Lunar New Year (Spring Festival), so that by the time the measure was imposed, more than 1/3 of the population had already left the city, to return to their ancestral homes across China (Chen et al., 2020). More of the city’s citizens had travelled around the world to take advantage of the 2-week holiday. By March 2020, the World Health Organization declared Covid-19 a pandemic. By then, more than a dozen countries, including the United States had reported cases of the disease within their borders and initiated international travel restrictions (Barba-Sánchez & Atienza-Sahuquillo, 2017; Santos et al., 2017).

The diffusion of the disease, following the standard S-shaped curve at a vastly accelerated rate because of modern air travel, had reached globally by May 2020. Those countries that were fortunate to escape the initial first wave, did not make it through the summer of 2020 unscathed (Beliaeva et al., 2020; Nissan et al., 2011).

While the mortality rate of Covid-19, at 2.2% globally, remains much below that of the 1918 Spanish Flu (10–20%) the combination of global trade and information networks has likely resulted in permanent re-orderings of consumption and production patterns. Scholars have estimated that the 1918 Spanish Flu pandemic led to loss economic wealth that lasted well into the 1980s (Almond, 2006). We expect Covid-19 to have similar long-term consequences. That said, this reordering of consumption patterns is likely to create new opportunities and resource pools for business venturing (Brünjes & Revilla-Diez, 2013), and innovation by individuals and organizations (Zahra, 2020).

## Absorptive capacity systems theory

Absorptive capacity refers to the knowledge stores a firm possesses that confers the capability to utilized externally acquired information (Cohen & Levinthal, 1990). It is a key ingredient in a firm’s ability to adapt, innovate, and respond to environmental change. The antecedents of absorptive capacity are prior related (to the innovation task at hand) knowledge, the structure of organizational communication, and the specific or general distribution of specialized knowledge within the organization. In this paper, we extend the notion of absorptive capacity to include other resources such as financial, human, and political capital, because these resources, just as knowledge capital, are part of the infrastructure necessary for adaptation.

The idea behind absorptive capacity goes back to Thompson (1967), who discussed the importance of resource buffers for organizational effectiveness. Organizational effectiveness is defined as the continuing ability to survive change without depleting the store of resources. Building on Thompson (1967), Bourgeois (1981) articulates the construct of organizational slack, which is defined as the financial resources that firms need to respond to environmental shocks. His contrasts organizational efficiency (the ratio of outputs over inputs) and organizational effectiveness, stating that the latter requires firms to operate inside the organizational efficiency frontier. Absorptive capacity extends the notion of organizational slack to include knowledge resources, some of which is embedded in individuals and others in collectives, as the means for adaptation and innovation (Cohen & Levinthal, 1990). The threads connecting Thompson (1967) to Cohen & Levinthal (1990) are environment change and the firm’s resources necessary for reacting and adapting to change. Based on the lineage of the term, we refer to buffering resources as absorptive capacity, even though the original usage of the term refers only to knowledge stores.

### Macroeconomic absorptive capacity

The macroeconomic effects of Covid-19 can be observed in the immediate shock to the dynamics of production and consumption. The pandemic triggered a four-part crisis, affecting risk and uncertainty in the socioeconomic order. First, the pandemic placed an unprecedent burden on health systems, which negatively affected the consumption and production of non-Covid-19 related health services such as cancer screening and elective surgical procedures (Kuckertz et al., 2020). Second, the infection control measures led to sudden (within a quarter) and dramatic slowdowns in domestic economies globally, ranging from 10% of GDP in Germany to over 20% in the United Kingdom. Economic contractions are well known to central banks around the world. However, the speed and size of the decline meant that central banks did not have time to develop new policy tools and so deployed traditional measures like quantitative easing that may be less appropriate.

Third, the proximate cause of the demand contraction was a public health shock. This demand contraction can be decomposed into two parts. An endogenous shock cause by the reduced consumption of certain goods and services, such as air travel and hotel rooms, and an exogenous shock caused by infection control measures such as lockdowns and capacity controls preventing production. By some estimates, the impact of social distancing measures resulted in a 15% decline in production over the 3-day period following their implementation (Deb et al., 2020). Fourth, the severity of the long-term impact is likely to be socioeconomic in nature, as we will later discuss.

We submit that Covid-19 is qualitatively different from previously studied macroeconomic shocks, such as the Haiti earthquake in 2010 or the 2011 Tohoku earthquake in Japan, with damage in excess of USD 210 billion,^2^ because the latter events were short lived and regionally confined. In those examples, supply chains, which represent the resource buffers of an economic system, were relatively intact and still able to support economic recovery efforts. In the Covid-19 pandemic, the global supply chain for major categories of goods and services was abrupted cut. Examples include the disruption of food supply chains due to lockdowns in farms and meat processing plants (Middleton et al., 2020), cancelling of elective medical procedures due to hospitals’ infection control policies (Negopdiev et al., 2020), decline in airline travel due to border closings and even restrictions on regional domestic travel in such places as Australia and China (e.g., Moloney & Moloney, 2020), and closure of movie theatres and restaurants due to occupancy restrictions (Ozili & Arun, 2020).

The shock to production and consumption unsurprisingly spilled over into the labor market. The size and speed of spillover has been historic. For the first time in more than a generation, the unemployment rate in the United States reached double digits, after recording the lowest rates across all demographic groups for several decades (Coibion et al., 2020a, b). This sudden and massive change in circumstances has created unanticipated but, in hindsight, knowable negative consequences for companies and individuals. Some of these consequences are likely to be long term, such as the reconsideration of business travel when teleconference meetings suffice.

### Microeconomic absorptive capacity

The pattern of demand shocks suggest that Covid-19 infection control measures have had industry-specific impacts, even though spillovers such as the reduction in demand for gasoline fuel are more general. The worse effects are most evident in the service-related industries, in which a large share belongs to small and medium size enterprises (SMEs).Examples include the near-instant collapse in demand for inbound travel and leisure services such as hotel rooms, and tourist attractions such as museums and theme parks (Ozili & Arun, 2020). Popular destinations in the United States experienced up to 90% shrinkage in inbound traffic in the first two weeks of the March 2020 lockdown order, hitting airline seats, hotel rooms, and restaurant covers (Suzumura et al., 2020). Airlines around the world parked between 50- 90% of available seat capacity, predicting that a return to full operating capacity could be years away due to continuing border control measures (Sun et al., 2020). Business travel, long the mainstay of airline profitability, is unlikely to return to full demand, given the continuing work-from-home (WFH) policies through at least the summer of 2021 (Bick et al., 2020), and the increasing acceptance of conducting business meetings via teleconference. These shifts translate into critical impact on tens of thousands of SMEs that compete in the travel ecosystem, from independently owned restaurants and cafes and tourism focused retail to ground transport operators and mom-and-pop accommodation establishments. Commercial real estate values extend their long-term decline as SMEs fail and corporations make permanent their work-from-home policies (Ling et al., 2020).

A systems view of the pandemic requires us move beyond atomistic considerations of individuals and firms toward the context in which they interact with other actors in the economy (Doern et al., 2016; Herbane, 2010). Similarly, entrepreneurship in a crisis is not a process that can be adequately described by the actions of single actors, as is customary in most research on entrepreneurship but rather the interactions among actors, industries, and regions, and their market and political coordinating mechanisms (Bailey & Breslin, 2020; Linnenluecke, 2017). To understand this process, we first have to acknowledge that the pandemic has led to step changes with long term consequences, in what used to be considered normal activities in daily life. For example, data reveals that routine health checkups that would have led to the early detection of cancer substantially declined during the pandemic, which may suggest higher incidences of advanced stage cancers in later years of life (Lange et al., 2020). Similarly, school closures may portend the loss of future income. By some estimates a 4-month closure of K-12 schools in the U.S. in the Spring of 2020 created 1.25 trillion U.S. dollars in loss lifetime future income for 76 million students (Psacharopoulos et al., 2020). In sum, these changes may have reduced a society’s absorptive capacity to innovate in future generations.

Second, SMEs do not generally have the free cash flows to weather sudden large declines in income. Mass SME failures following catastrophes such as Hurricane Katrina in 2005 (Runyan, 2006), and the Lehman Brothers collapse in the 2008 (Chow & Dunkelberg, 2011) can have long lasting effects on the local communities that depend on them. Some environmental shocks can create such a high level of uncertainty that decision makers are unable to assign an expected value to various outcomes. In these instances, incumbent firms have to manage the dual challenges of reconfiguring existing resources to maximize their productive use or innovating to find new sources of growth. Both activities require firms to be fully engaged in responding to the crisis. Yet, we know that decision-making under conditions of uncertainty is beset by inertia, which driven by the decision makers’ knowledge filters (Acs & Plummer, 2005; Acs et al., 2013). The strength of knowledge filters is the consequence of a firm’s absorptive capacity at time of the crisis. Firms with greater absorptive capacity will regard the crisis is an opportunity to pull ahead of the competition. Greater stores of prior knowledge mean weaker knowledge filters and are thus better positioned to identify and exploit the market spaces left by the departure of existing firms or created by new unmet needs created by the pandemic. For others, the uncertainty represented by the crisis will discourage experimentation, particularly because the necessary capital can only be required at a higher discount rate to reflect the higher risk to investors.

Crises also create opportunities for new entrants unhobbled by existing resource commitments and organization structures resistant to change. In the language of absorptive capacity, in economies dominated by SMEs knowledge spillovers from existing sectors create opportunities for entrepreneurs with the appropriate knowledge filters. Similarly, regions that exhibit a high level of systemic support for entrepreneurship (i.e., minimal legal requirements for business registration, abundant risk capital and debt funding, deep knowledge capital in the form of post-secondary education, inviting cultural and living environments, robust telecommunications infrastructure, and so on) in the pre-crisis period would have the necessary absorptive capacity for startups to exploit the shocks generated by the pandemic (Bishop, 2019; Bishop & Shilcof, 2017; Williams & Vorley, 2015). It is therefore unsurprising that new firms continue to be created even during crises.

In conclusion, the emergence of SMEs in market-based economies fill gaps left by industrial upheaval is an inexorable process. However, *which* SMEs and in *what* sectors will survive the shock remains the subject of speculation. For example, a 50% or 75% restriction in available capacity meant that some full-service restaurants and legacy airlines were better off staying shut. If sufficient numbers of establishments make this choice, the ripple effect on the upstream supply chain becomes significant. On the other hand, takeout restaurants and discount airlines are probably resistant to, and may even thrive on, the shifts in consumption caused by the pandemic.

Whether the recovery of the microeconomy will be quick is unclear because every productive sector has been affected and the results of the fiscal interventions are still unfolding. First, the deployable human capital for innovation has been impacted by disease or infection control measures. Second, government interventions to support household consumption run the risk of redirecting productive capital away from innovation and new venture creation. Finally, if vaccines and effective therapies take too long to reach the general population, new consumption patterns may become entrenched. For example, the reversal of recent trends in urban gentrification is likely to shift consumer demand from the cities back to the suburbs or exurbs while consumers habituate on distance learning, telemedicine, and online shopping, all of which change the opportunity landscape for SMEs.

### Individual absorptive capacity

The individual actor is the smallest unit of analysis in a socioeconomic system. We know that the Covid-19 pandemic has had a positive effect on self-employment. When unemployment increases, the number of the self-employed also increase, especially when short term unemployment supports cease. The Covid-19 crisis has led to the uptick in, for example, delivery drivers for e-commerce services, and brokers to connect the supply and demand for personal protective equipment (PPE). Similarly, there is ample empirical evidence showing that entrepreneurial activities increase following disasters like earthquakes (Williams & Shepard, 2016). However, self-employment and entrepreneurship are not the same thing and absorptive capacity is more likely to explain the emergence of entrepreneurship than the increase in self-employment.

According to the literature, self-employed persons are focused on reducing the risk to income whereas entrepreneurs are focused on the opportunity created by uncertainty. In the entrepreneurship literature, risk (known-unknowns) is distinct from uncertainty (unknown-unknowns). Risk can be hedged if one knows the shape of the distribution (mean and variance) of the outcomes, whereas uncertainty cannot be hedged but instead resolved only by effectuating the idea (Knight, 1921). Hence, the self-employed start businesses to replace lost or at-risk income. The overwhelming part of new business creation caused by the crisis can be regarded as employment substitution. The latter, which forms a smaller part of the population of the self-employed, are motivated by uncertain but significant future profit, and may be driven by self-efficacy, curiosity and creativity and overconfidence as a problem solver.

Consequently, an exogenous shock like the pandemic affect entrepreneurs and self-employed persons differently. The self-employed switches to self-employment as a means of protecting present income. The entrepreneur is focused on the reordering of means-ends brought about by the pandemic in order to exploit perceived opportunities. For example, a self-employed person may start making masks to sell online as a means of earning an income after being laid off, whereas the entrepreneur may decide that masks may become a permanent feature of face-to-face interactions and therefore proceed to design more comfortable masks that enhance, rather than inhibit, speech and facial communication.^3^

The functions that characterize entrepreneurship are discovery, evaluation, and exploitation of business opportunities (Shane & Venkataraman, 2000). According to the theory of knowledge spillovers, entrepreneurs recognize opportunities based on existing knowledge stores, and previous experiences in related domains. Accordingly, ideas and knowledge created in one organizational context serves as a source of inspiration for the recognition of entrepreneurial opportunities in other contexts (Caiazza, 2016; Caiazza et al., 2020). These knowledge filters can be seen as the absorptive capacity responsible for the variation of new ideas in an economy, and also explains why incumbent firms may not adapt as quickly or radically as entrepreneurs. Entrepreneurial initiative is the conduit facilitating the spillover and commercialization of that knowledge. In turn, entrepreneurial opportunities are generated not just by investments in new knowledge and ideas, but in the propensity for a subset of those opportunities to be fully pursued and commercialized by entrepreneurs (Audretsch et al., 2020; Belitski et al., 2019a, b; Caiazza et al., 2015). Hence, the combination of entrepreneurial attention, personality, knowledge, and experience held by individual actors is the basis for new value creation.

Besides the impact on personal income, the sudden loss of employment at a global scale has severely affected the mental health for many individuals. Suicide rates (John et al., 2020), alcoholism, opioid overdose-related deaths, domestic abuse, and other indicators of mental health decline are measurably higher (Cullen et al., 2020) during the pandemic. For the millions of individuals, especially youth, who found stable jobs in 2019 after long bouts of unemployment, the impact on mental health in 2020 has been particularly acute (Bartelink et al., 2020), and potentially long lasting.^4^ In effect, individuals who are newly diagnosed with mental health conditions add to the rolls of the healthcare consumer while taking away from the rolls of the value creator. The impact on mental health has important consequences for entrepreneurs.

Recent research on mental health and entrepreneurship (Nicolaou et al., 2020; Torrès & Thurik, 2019) have revealed important relationships that cannot be ignored when considering the consequences of the pandemic infection control measures. The documented increase in Covid-19 related depression, if persistent, has important consequences for SMEs that need to rehire as the economy recovers. Individuals suffering from mental health are usually less prepared to enter the job market or keep the jobs that they have (Bartelink et. al., 2020), shrinking the supply of labor resources just when it is needed for recovery.

## Discussions

### Absorptive capacity and innovation

According to Schumpeter, creative destruction describes the process of industrial change caused by technology substitution. This process continually revolutionizes the economic relationships between producers, consumers, and institutions by forcing out less adaptable incumbents to make room for more innovative entrants. From a systems perspective, Schumpeterian innovation is one of endogenous change. But in the case of Covid-19, the shock is exogenous to the system of value creation. What we see in the pandemic is individuals and firms being forced to act entrepreneurially because of the lack of viable alternatives (Amit & Muller, 1995). For example, white tablecloth restaurants adapting their service models to food delivery is not the consequence of a proactive marketing strategy but an operational reaction due to infection control measures. The consequences for their business models, with high furniture, fixture, and equipment (FFE) costs, are not sustainable but could point the way toward new service models such as distributed kitchens.^5^

A large enough shock capable of destabilizing an existing equilibrium leads to means-ends reordering that create new opportunities for entrepreneurs. Then, innovation emerges to fill the voids in a destabilized system of economic relationships (Agarwal & Audretsch, 2020). In a global crisis, this dynamic is played out across multiple industrial sectors and geographic regions that are connect by trade flows, and ultimately responsible for the emergence of new industries and firms (Bishop, 2019).

Probably, the best example of innovation across multiple domains during Covid-19 is the rapid development of the vaccine, first deployed at the end of 2020 by BioNTec, an academic spin-off from the University of Mainz, Germany in cooperation with Pfizer. Other vaccines have quickly followed, mostly developed by university-industry partnerships such as AstraZeneca and Oxford University (UK) or Curveac (University of Tuebingen, Germany) and Bayer (Lurie et al., 2020). There are at least 2 well known aspects of this story. First, is the speed at which the vaccines were develop would not have been possible without prior knowledge or technological absorptive capacity. The search for a vaccine began in February 2020 after the publication of the genetic sequence of the virus. It was known at the time that the virus is an RNA-based pathogen that infected host cells via a spike protein on the surface of the virus surface. Simultaneously, a novel vaccine discovery platform using messenger ribonucleic acid (mRNA) was in development (Monslow et al., 2020). In a study published more than 10 earlier, CEO and founder of CureVac Ingmar Hoerr first showed that mRNAs can provide an attractive alternative to peptide and DNA-based vaccines. This laid the foundation for the first mRNA-based immunotherapy (Hoerr et al., 2000) and pointed the way to challenging the 70-year-old technology of growing viruses and deactivating them for use as vaccines. However, development had stalled because there was never a market large enough to justify the costs of the new approach when an existing one was sufficient. It was not until Covid-19 that the economics of the mRNA platform received its first full test, with the first Covid-19 vaccine authorized for use a mere 10 months from bench experiments (Lurie et al., 2020).

Second is the reordering of institutional roles and expectation that created the absorptive capacity for the exploitation of the new vaccine technology. This institutional innovation is illustrated in the way the U.S. White House, through Operation Warp Speed (OWS) de-risked vaccine development by pre-purchasing a hundred million doses of the vaccine, turbo-charging the EUA provision in FDA regulations, coordinating scientific development, pre-positioning distribution logistics, and accelerating regulatory review to shrink the time from discovery to bedside (Slaoui & Hepburn, 2020).

The technological and institutional innovations related to the Covid-19 vaccine program are probably the most lasting legacy of the pandemic response because they accelerated pharmaceutical development in ways not imagined and reset future expectations for what is possible. Taken together, this process nicely illustrates the triple helix model of negotiated interactions among academia, industry, and government. This model explains how new organizational forms, such as incubators, science parks or corporate venture capital that underpin the translation of science to products work to foster the creation of new firms to solve public problems with private means (Etzkowitz, 2002). The triple helix is a convenient way to understand the government’s role, which is to create the conditions for innovation by de-risking the process for private firms that would otherwise face an economic and logistical barrier to actualizing their ideas. Operation Warp Speed turned what would have traditionally been a big government program into a negotiated interaction between big pharma (clinical trials, regulatory management, and production), biotechnology (bench science), academia (clinical trials and bench science), government (financing, regulation) and the military (logistics and distribution). Each partner in the value network plays specific roles and is coordinated by a common interest.

For triple helix regimes to work, the absorptive capacity of the partners must match. Weak regulatory management coupled with accelerated vaccine development might lead to products that further threaten human health or weak logistics networks coupled with well-regulated vaccine development will not have the scale of impact on population health that makes a difference to the disease course. Triple helix regimes follow a deliberate process to balance capability portfolios to exploit entrepreneurial initiative in service of a public good.

In addition to vaccine development, innovations could be seen everywhere in society, including ideas to double the capacity of mechanical respiratory ventilators with 3-D printed splitters that could maintain the correct positive-pressure (Clarke, 2020). More prosaically, SMEs making and marketing personal protective equipment such as boutique masks and transparent masks have become a new category of business in the industrial landscape.^6^ Industrial reordering is evident in the speed at which automobile factories were reengineered to make sophisticated ventilator machines^7^ or high-end whiskey distilleries repurposed to make hand sanitizers and surface disinfectants.^8^ This ‘whole-of-society’ response was possible because innovators had existing technical knowledge and production capabilities that could be repurposed.

We also witnessed innovation in the way therapies were rapidly tried through off-label prescribing by doctors, using medicines indicated for malaria, inflammation, asthma, rheumatic arthritis, and other seemingly unrelated diseases whose symptoms present in some Covid-19 patients (Rogosnitzky et al., 2020). These innovations by healthcare providers (rather than pharmaceutical companies), such as the use of convalescent plasma, were given EUA on the basis of preliminary data at lower thresholds of efficacy but with similar standards of safety. While off-label prescribing is a common practice in medicine, the scale at which this approach has been used, combined with clinical trial protocols, is unheard of. At its peak there were more than 200 investigations of various therapies for Covid-19 underway around the world, many of which was focused on repurposing known drugs (Lam et al., 2020). The liberal use of the EUA is itself an institutional change that demonstrated what is possible by government agencies.

Finally, the closure of schools from pre-school to universities around the world, have led to structural changes in the way education is delivered in developed countries. Prior to the pandemic, online education represented a small share of formal degree university programs, and an insignificant fraction of primary and secondary education. During the lockdowns, we saw the explosion of digital education (EdTech) at every level, from early years to adult learning. As a result, EdTech investment grew by 15% in 2020, representing 7.6 billion U.S. dollars of market value. Since then, the world’s startup hubs have been developing an ever-growing number of EdTech solutions that find their usage in various places from universities and schools, to remote employee onboarding, and upskilling in the workplace. In 2020, London-based EdTech companies raised a total of 124 million U.S. dollars in VC investment, ahead of Paris with 92 million U.S. dollars and Berlin with 67 million U.S. dollars, making London’s EdTech ecosystem the largest in Europe, with an estimated market value of 3.4 billion U.S. dollars. Worldwide, China dominates investments in EdTech, with Beijing in first place and Shanghai in third, while San Francisco and Bangalore come in at second and fourth, respectively.^9^ Yet, whether growth in EdTech means greater societal absorptive capacity to innovate is unclear as the effectiveness of online education in K-12 may never be fully understood for at least 2 decades.

### Policy responses as absorptive capacity

The shock to discretionary consumption brought on by social distancing led to fiscal countermeasures to slow the spiral toward structural recession. The concern among policy makers is the contagion of corporate bankruptcies spiraling across the economy and hitting SMEs with the least financial ballast. By summer of 2020, nearly 100 large companies with more than 100 million U.S. dollars in debt filed for Chapter 11 bankruptcy protection, according to the American Bankruptcy Institute.^10^ According to a U.S. Chamber of Commerce poll, about 43% of small businesses would close permanently in the final months of 2020.^11^ Some of these are pre-emptive closures but most are due to unserviceable debt. Hence, fiscal measures to slow the rate of bankruptcy could be interpreted as attempts to create absorptive capacity.

As stated earlier, the impact of the crisis has been most keenly felt in the service sector where SMEs dominate. In fact, it is the concern over the SME labor market that formed the basis for the U.S. Federal Government’s Cares Act and Paycheck Protection Program,^12^ since SMEs account for the largest share of employment in the U.S. Yet, the return to full economic activity, in spite of the much touted ‘V’ shape recovery remains in question for SMEs because each report of a Covid-19 cluster or new variant causes local governments to reimpose infection control measures that usually hit SMEs the hardest. For example, restaurants that invested capital in outdoor dining may not recover these investments due to the rolling lock downs that have now extended to outdoor spaces.^13^ Uncertainty increases the costs of investing and many service sector SMEs have chosen to permanently close rather than wait for a stable recovery (Gourinchas et al., 2020a, b).

### Reflections on the fiscal interventions

Policy analysts have compared the Covid-19 crisis to previous financial crises such as the 1997 Asian market crisis, the 1999 Latin American crisis, and, most recently, the 2007 Great Recession in Europe and the United States.^14^ The implication being that the policy interventions in those crises can served as models for Covid-19. Indeed, the idea of prime pumping the economy to forestall a recession achieved bipartisan acceptance (the Cares Act) due to this line of thinking. We believe that such comparisons are mistaken. By not accounting for the *causes* of the crisis before deploying the same policy instruments, such as a 3 trillion U.S. dollar cash infusion, we may prolong the road to recovery by propping up already weak companies, creating the conditions for uncontrolled inflation in the future, or redirecting productive (innovation) capital to unproductive (consumption) uses when the latter is unnecessary.

Fiscal policy interventions lag the events that trigger them (Iyke, 2020). Generally, central bankers are leery of ‘over-correcting’ and thereby extending the crisis. Covid-19 changed this calculus. Not only was the U.S. 2020 Cares Act preemptively passed in March 27, before the full effects of social distancing were felt and only 2 weeks after the pandemic was declared by the WHO, the quantum of the package, at 2.2 trillion U.S. dollars, was breathtaking. The speed, timing, and size of the rescue package was designed to replace the anticipated income loss, and to help SMEs keep their employment rolls. Many countries followed the U.S. example, though to a lesser quantum. It was believed that keeping workers on the employment rolls positively affected consumer confidence and created the conditions for faster recovery. Hiring and onboarding costs of new employees are not trivial for SMEs. These costs are avoided by keeping current employees on the job even if they not working at capacity. More importantly, preemptive fiscal intervention is believed to mitigate a crisis in consumer confidence, which would allow SMEs to retain their investments in productive capital. Even major airlines, some of which parked up to 95% of their fleets, did not initially permanently reduce capacity, choosing instead to use their loan facilities to stay in operation while waiting out the recovery (Budd et al., 2020).

Scholars and policy experts will continue to debate the viability of Cares Act-type interventions for years. Yet, politicians are calling for even more public spending in the belief that it will mean faster recovery.^15^ However, the initial Cares Act, and its copycats around the around, was possible because of the pre-pandemic near-zero interest rates, historic low unemployment, and high productivity growth. This combination of factors is unique and unlikely to be repeated, especially if the general cost of business, such as energy prices, spikes. More critically, because the Covid-19 recession was not caused by a secular contraction of credit or consumption, adding more liquidity to the economy is unlikely to trigger the behavioral changes needed for recovery. For example, no amount of liquidity will make people fly in planes, eat in restaurants, or go to cinemas if they continue to feel unsafe or do not want to be responsible for spreading the virus.^16^ Prime pumping an economy already consuming at the productivity frontier in the pre-pandemic era is, by definition, inflationary, which *would* trigger a structural decline in consumption.

Policy interventions of this magnitude carry two lessons. While decisive preemptive action can mitigate a confidence crisis in consumers and producers, the causes of the crisis have to be central to the policy design; and*not* over-correcting remains a valid and critical policy goal. In the case of Covid-19, the Cares Act broke mental models few thought possible. The apparent willingness by policymakers such as the Federal Reserve (the U.S. Central Bank) and the European Central Bank, whose primary mission is to control inflation, to ignore the dangers of public debt is notable. The global consensus, that future obligations should be ignored to relieve current pain,^17^ is a mindset change associated with Covid-19. Save for a few voices,^18^ there has been little concern for the consequences and whether a Rubicon has been crossed or if this is a unique case remains to be determined.

Finally, we note that macroeconomic policy interventions are, by definition, blunt and inefficient instruments to achieve individual and regional outcomes. The pandemic did not hit every region of a country equally nor at the same time. An emerging story of the Cares Act are the inefficiency (funds not disbursed or disbursed too slowly), and fraud (individuals and companies applying for funds that they were not entitled to) in the program^19^ plaguing the program. Of course this was anticipated because the policy goal was supporting individuals and SMEs rather than minimizing waste.^20^ Yet, one could contemplate finer-grained ways to direct liquidity, perhaps by acting as a credit backstop for community banks at the local level rather than making direct payments.

### Reflections on SME recovery in the post-pandemic period

There have been many analyses using mobility data to gauge the impact of social distancing measures on local consumption. For example, mobility data showed that the number of visits to retail stores, as measured by credit card spending, and mobility data declined 95% during the first two weeks of the lockdowns in the U.S. (Huang et al., 2020). Although retail patronage has recovered slowly since, and partially due to lock down measures being localized in such places as California, one of the most enduring effects of the social distancing policies is the change in consumption patterns; from cinema-going to video streaming, international travel to staycations, restaurant outings to home cooked or home delivered meals, retail shopping to online shopping, and so on.^21^ Data from the 2020 Thanksgiving shopping season showed that while bricks and mortar stores experienced much lower spending, online shopping held its own.^22^ These shifts disproportionately affect SMEs since they seldom have the means to mitigate losses in local markets by tapping distant ones.

Preliminary evidence from cities such as Wuhan, ground zero for the pandemic, suggest that mobility and economic activity do not respond quickly to reopening. Consumers may have habituated to a new way of living and may take time to regain trust in the safety of the places they used to frequent. Past research on product recalls, and the example from the 1918 Influenza epidemic suggests that consumer schemas, once formed, are difficult to change (Crosby, 2003). The complete return of demand to local markets may never occur. More importantly, the harmful consequences of public health interventions have not been fully explored – we have only begun to understand the effects on childhood education, mental health, and undiagnosed chronic diseases and cancer (Itai et al., 2020), suggesting that the shape of full economic recovery is unclear.

We know that recovery on the production side will involve, among other things, the reconstruction of supply chains disrupted by the pandemic, rehiring of labor, and establishment of new lines of credit. For example, the sudden loss of work-in-process inventory in a restaurant due to lockdown orders represents anuncoverable loss of working capital that negatively affects its credit rating, and for which the Cares Act, designed to protect labor, did not address. Re-hiring the workforce proved challenging for some SME after the first locked down period, in part because of some permanent shift from employment to self-employment.^23^ The Paycheck Protection Program in the United States, as is the case in other parts of the works, incentivized the labor force to stay at home, a goal of the program, that may have kept some individuals out of the labor market longer than anticipated. Others may be slower to return to employment because of the mental health challenges we discussed earlier and yet others may have secured alternate employment and unable to return to former employers. These barriers will delay the return to full productivity of SMEs that depend particularly on skilled labor.

Notwithstanding the widespread agreement over the economic consequences of Covid-19, we note the positive effects on demand for certain classes of goods and services. Specifically, the demand shock has been almost entirely confined to discretionary consumption. Caloric intake has not declined (Rolland et al., 2020), and some categories such as alcohol have increased (Ramalho, 2020) due to the lockdowns. Hence, the demand shock might be better described as a reordering of consumption rather than a decline in consumption. For example, employers’ work-from-home policies have boosted demand for office furniture,^24^ information technology and communications equipment and software, online shopping, streaming video and online entertainment, and home delivery services (Dannenberg et al., 2020). These patterns, combined with the rapid recovery of the U.S. equity market,^25^ suggest that overall consumer demand remained healthy throughout the period. More generally, the extended lockdowns in some countries may have permanently changed consumer preferences and habituated behaviors to, for example, the use of online technology. In addition to education delivery, the widespread adoption of online business meetings, family events and even church going has made individuals increasingly comfortable with this type of interaction. The longer the pandemic lasts the more likely these habits become the norm, creating new opportunities for entrants. Prominent examples including online gaming. The increase revenues experienced by e-gaming companies during the pandemic suggest the participation of new demographic groups, leading to the entrance of complementary technologies and startups in virtual animation, cloud storage, decentralized computing power, and specialized social networks. This dynamic suggests opportunities for startups.

## Conclusions

The purpose of this paper has been to articulate an absorptive capacity-based systems perspective of the effects of the Covid-19 pandemic on SMEs. As with all theories, there are limits to which a point of view can be applied to different geographies, industries, and cultures. For example, the political and legal institutions of economy (Common Law versus Civil Law) determine the power of government to impose restrictions on freedom of movement. In an innovative use of its public health authority, the U.S. Centers for Disease Control (CDC) released an advisory against tenant evictions, on the premise that occupants who end up homeless risk Covid-19 exposure.^26^ Such institutional innovations are more likely in laisse faire political systems since they arise from the actions of atomistic policy makers. In centralized political systems, institutional innovation is less likely.

In this paper, we have taken the position that the scale and reach of the Covid-19 pandemic is such that the traditional view of effective crisis responses as a return to a pre-crisis equilibrium, especially for SMEs, needs to be re-examined. We have provided reasons why this may be the case but are open to the possibility that status quo bias and inertia of the individual decision maker could invalidate our view.

In terms of future research, our paper suggests that researchers need to examine each crisis as a unique event. Knowing how SMEs behave in one crisis does not necessarily generalize to how they will do to others. A volcanic eruption is a singular event with well-defined outcomes whereas a fast-moving pandemic produces policy responses that are likely create unanticipated side effects, which may be worse than the triggering event. In effect, the more multidimensional a crisis the more unique the combination of proximate causes and effects.

Finally, whether the lessons learned from this crisis is applicable to future crises will depend on how policymakers interpret the effectiveness of their interventions. If policymakers remain open to innovation, the knowledge gained from this crisis would represent the absorptive capacity for dealing with future crises. If policymakers see there their responses as optimal, then lessons learned from this crisis could turn into a straitjacket in dealing with future crises.

## Footnotes

1 The speed of viral transmission guarantees that these numbers are out of date the moment they are quoted. For the latest figures, please refer to https://coronavirus.jhu.edu/map.html (12/23/2020).

2 See https://www.munichre.com/en/risks/natural-disasters-losses-are-trending-upwards.html#-1624621007 (accessed: 8/13/2020).

3 https://hub.jhu.edu/2020/10/19/clearmask-sells-11-million-masks-worldwide-during-pandemic/ (accessed: 1/4/2021).

4 https://www.wsj.com/articles/in-france-the-young-lose-their-jobs-to-coronavirus-11598888747 (accessed: 12/14/2020).

5 https://techcrunch.com/2018/10/07/the-next-big-restaurant-chain-may-not-own-any-kitchens/ (accessed: 1/4/2021).

6 https://www.wfaa.com/article/news/local/garland-apparel-manufacturer-makes-quick-shift-to-ppe-production/287-7bf0b040-82d6-4a80-a5ac-f3d6b216eaa0 (accessed: 12/14/2020).

7 https://nypost.com/2020/04/08/gm-to-make-30000-ventilators-to-help-us-fight-coronavirus-pandemic/ (accessed: 12/14/2020).

8 https://parade.com/1011922/jerylbrunner/distilleries-making-hand-sanitizer/ (accessed: 12/14/2020).

9 London leads European EdTech revolution, download: https://www.webwire.com/ViewPressRel.asp?aId=263682 (accessed: 11/30/2020).

10 https://www.bloomberg.com/news/articles/2020-04-10/record-bankruptcies-predicted-in-next-year-as-unemployment-soars (accessed: 12/14/2020).

11 https://www.uschamber.com/report/july-2020-small-business-coronavirus-impact-poll (accessed: 12/14/2020).

12 https://home.treasury.gov/policy-issues/cares (accessed: 12/14/2020).

13 https://www.usatoday.com/story/news/nation/2020/11/13/covid-restrictions-state-list-orders-lockdowns/3761230001/ (accessed 12/14/2020).

14 https://www.brookings.edu/blog/up-front/2020/03/03/what-should-a-fiscal-response-to-a-covid-19-outbreak-look-like/ (accessed: 12/14/2020).

15 https://www.wsj.com/articles/the-economy-needs-a-little-more-ppp-11607469632 (accessed: 12/14/2020).

16 Early results from the Cares Act suggest that individuals used the money to pay down debt or increase savings, and not to fund consumption (Coibion, Gorodnichenko, and Weber, 2020b).

17 https://www.wsj.com/articles/in-europe-monetary-policy-is-all-about-giving-banks-free-money-11607621401 (accessed: 12/14/2020).

18 https://www.iisd.org/articles/financing-covid-19-stimulus (accessed: 12/14/2020).

19 https://www.wsj.com/articles/ppp-was-a-fraudster-free-for-all-investigators-say-11604832072 (accessed: 12/14/2020).

20 https://www.wsj.com/articles/sba-to-face-big-challenges-ensuring-coronavirus-loans-arent-misspent-11588094140 (accessed: 12/14/2020).

21 https://www.wsj.com/articles/a-new-setback-for-big-cities-as-return-to-the-office-fades-11606818601 (accessed: 12/14/2020).

22 https://www.wsj.com/articles/black-friday-deals-become-tamer-as-covid-19-alters-sales-strategies-11606473001 (accessed: 12/14/2020).

23 https://www.wsj.com/articles/in-the-covid-economy-laid-off-employees-become-new-entrepreneurs-11605716565 (accessed: 12/14/2020).

24 https://www.furnituretoday.com/research-and-analysis/covid-19-creating-opportunities-in-home-office-furniture/ (accessed: 12/14/2020).

25 https://www.reuters.com/article/us-usa-stocks-dow30000-psychology-analys-idUSKBN2842OJ (accessed: 12/14/2020).

26 https://www.federalregister.gov/documents/2020/09/04/2020-19654/temporary-halt-in-residential-evictions-to-prevent-the-further-spread-of-covid-19 (12/30/2020).
